# Supplementary material for: Patterns of Intron Gain and Loss in Fungi
Source: PLoS Biol. 2004 Nov 30;2(12):e422. doi: 10.1371/journal.pbio.0020422 (PMC532390; doi:10.1371/journal.pbio.0020422)
Supplement: Table S1 — Also available at http://genes.mit.edu/NielsenEtAl/. (4.3 MB ZIP). [file pbio.0020422.st001.zip › NielsenEtAl/html/1419.html]

AN6158.1.NCU01320.1.MG06907.1.FG01897.1


```
 CLUSTAL W (1.82) Multiple Sequence Alignments - Introns Inserted


Sequence 1: MG06907.1	124 aa
Sequence 2: FG01897.1	149 aa
Sequence 3: NCU01320.1	148 aa
Sequence 4: AN6158.1	163 aa
Alignment Length: 168 aa
Number Identitical Residues: 36 aa
Alignment Score (without introns) 2219


MG06907.1 	MSAAITLTLPGDYG2YVLLAAASSF-FLQTYHMVLSAKARKESGLMYPIAYAESQVAAK-
NCU01320.1	--MAITLTLPDEYG2YVLLATVSTF-FANSFHSINTGRQRKAAGVKYPLAYAPQEVAEK-
FG01897.1 	--MSLVLELPAEYG2YVLVAATSTF-FINTLHVVLTSKARKQSGLKYPIPYASNDLAEK-
AN6158.1  	---MVEISIPANYG2YAIAVSLGAIPVLGFIHGVLVGSFRKAAGVPYPHAYASIEQCKAN
          	    : : :* :** *.: .: .::..    * :  .  ** :*: ** .**  : .  .

MG06907.1 	~-------------DPKAYAFNCA~QRAQTNFTEN0LTPFLGALLIAGLKYPIPSAGLGA
NCU01320.1	~-------------DPKAFAFNCA1QRAHANFTEN~LTPAIGAMLIAGLKYPVLAGALGG
FG01897.1 	~-------------NAEAYKFNCA1QRSHANFTEN~QISFLGALLISGLRYPVASAVLGA
AN6158.1  	0IVFQERVLNDVCEQPKAYKFNCA~QRAHGNFLEN~APQTMLSILVAGVKYPEAAAGLGA
          	    ..   .. ..:.:*: **** **:: ** **     : ::*::*::**  :. **.

MG06907.1 	GWVFSRFIYASGYTTFG-PKGRG----------V------------------
NCU01320.1	LWSLTRVLYTIGYTKKG-PQGRTKFGIASSLSLLALKLMAAYTAVQIAFH--
FG01897.1 	GWAASRVFYAIGYSAGG-PQGRMGGSIGSALCDVSLKFMAAYTSIMYAMGN-
AN6158.1  	AWVVLRTLYMLGYIYSDKPNGTGRYN--GSLYLLAQAGLWGLSAFGVAKDLM
          	 *   * :*  **   ..*:*    .  .:   ::     . ::   :
```
